# Identification of Primary Hyperoxaluria Type III by Gas Chromatography/Mass Spectrometry-Based Urine Metabolomics

**DOI:** 10.3390/metabo16040278

**Published:** 2026-04-19

**Authors:** Tomiko Kuhara, Morimasa Ohse, Tatsuya Fukasawa, Koichi Maruyama, James Pitt

**Affiliations:** 1 Japan Clinical Metabolomics Institute, Kahoku 929-1174, Japan; jcmi-ohse@heart.ocn.ne.jp; 2 Anjo Kosei Hospital, Anjo 446-8602, Japan; fukasawa@kosei.anjo.aichi.jp (T.F.); maruyama@aichi-colony.jp (K.M.); 3 Victorian Clinical Genetics Services, Murdoch Children’s Research Institute, Melbourne 3052, Australia; james.pitt@vcgs.org.au

**Keywords:** diagnosis, *HOGA1*, kidney stone disease, metabolomics, primary hyperoxaluria type 3, hereditary urolithiasis, diagnostic criteria

## Abstract

**Objectives:** Primary hyperoxaluria type III (PH3) causes kidney stones in children and adults. Gas chromatography/mass spectrometry (GC/MS)-based metabolomics has been applied to study patients with primary hyperoxaluria types I and II, 2,8-dihydroxyadenine lithiasis, and xanthinuria types I to III. This study was performed to verify the usefulness of this technique for the diagnosis of PH3. Specifically, we evaluated an 8-month-old infant with recurrent kidney stones. **Methods:** GC/MS-based metabolomics was performed on spot urine samples using initial urease pretreatment without fractionation. **Results:** Metabolomics revealed increased levels of 2,4-dihydroxyglutarate and 4-hydroxyglutamate. No simultaneous elevations of these two critical biomarkers were observed in other patients, except for one case of PH3 confirmed by the identification of *HOGA1* mutations. A moderate increase in 4-hydroxyglutamate has been observed only in cases of primary hyperammonemia, in which analytes such as orotate, uridine, glutamine, or proline, but not 2,4-dihydroxyglutarate, are biomarkers, thus distinguishing PH3 from primary hyperammonemia. **Conclusions:** GC/MS-based urine metabolomics enables the rapid screening and chemical diagnosis of PH3 and other congenital anomalies that cause urolithiasis. This technique can also be used to monitor disease progression, as patients with PH3 benefit from long-term follow-up, particularly when transitioning from childhood to adulthood. The timely identification of patients with hereditary urolithiasis is crucial. To address this, a discussion was had about the current diagnostic criteria.

## 1. Introduction

**Figure 1:**
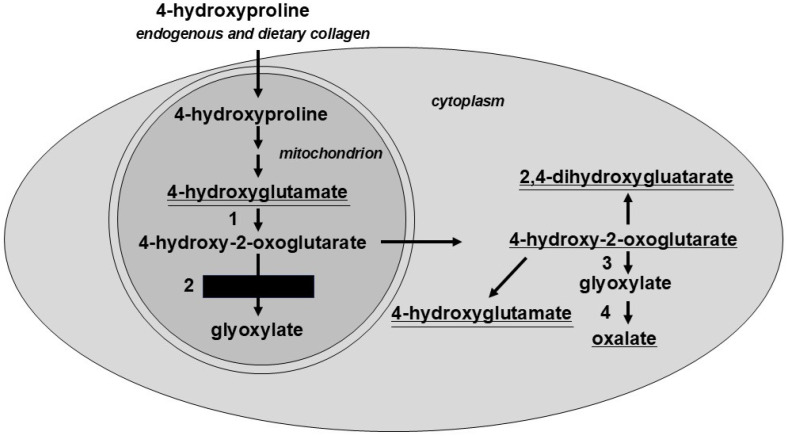
Metabolic pathway for 4-hydroxyproline, showing the enzymatic block (a black square) in primary hyperoxaluria type 3 and proposed metabolic re-routing mechanism [6]. Enzymes are as follows: (1) aspartate aminotransferase; (2) 4-hydroxy-2-oxo-glutarate aldolase; (3) unidentified aldolase; and (4) lactate dehydrogenase. Principal PH3 urine metabolites that exit the cell are underlined, two of which are double-underlined and can be used as reliable biomarkers.

Primary hyperoxaluria (PH) is a hereditary form of urolithiasis characterized by oxalate accumulation in the kidneys. PH3, the most recently identified PH subtype, results from defects in the mitochondrial enzyme 4-hydroxy-2-oxoglutarate aldolase (HOGA) in the 4-hydroxyproline catabolic pathway [1]. The causative gene, *HOGA1*, was identified in 2010 [2,3]. Timely identification and differential diagnosis of patients with suspected PH are crucial for preserving renal function [4]. Recent global genetic prevalence estimates of primary hyperoxaluria are greater than previously reported, suggesting that a large number of individuals who are at risk for PH symptoms remain undiagnosed [5]. The observation of increased urinary 4-hydroxyglutamate (4HGlu) in patients with PH3 has enabled metabolite-level diagnosis, using electrospray ionization tandem mass spectrometry [6]. Woodward et al. used liquid chromatography tandem mass spectrometry targeting 2,4-dihydroxyglutarate (2,4-DHG) for PH3 screening [7]. Both 4HGlu and 2,4-DHG are stable and are considered critical biomarkers for PH3, as shown in Figure 1.

Shoemaker et al. reported that the urease-pretreatment of urine and without extraction, followed by gas chromatography/mass spectrometry (GC/MS), enables simultaneous evaluation of the inborn errors of metabolism (IEM) of organic acids, amino acids and carbohydrates [8]. This approach was further expanded to target purine, pyrimidine, and nucleosides [9] to allow for simultaneous chemical diagnosis of IEMs in primary hyperoxaluria type I (PH1) and type II (PH2) [10], as well as 2,8-dihydroxyadenine lithiasis [11,12] and xanthinurias [13]. Chemical diagnosis of PH3 using GC/MS has not been widely applied for screening or confirmation in large cohorts of patients. GC/MS-based metabolomics is expected to be a suitable investigative technique, given its capacity to simultaneously detect a range of critical biomarkers of renal stone diseases due to IEMs, covering a range of chemical classes, such as organic acids including highly polar ones, amino acids, purines, pyrimidines and creatinine.

We present a case of a Japanese 8-month-old infant with PH3. GC/MS-based metabolomics was carried out for spot urine collected at 8 months 26 days of age. This study was performed to illustrate the utility of GC/MS-based metabolomics in screening for and diagnosing PH3.

## 2. Materials and Methods

### 2.1. Patient

**Figure 2:**
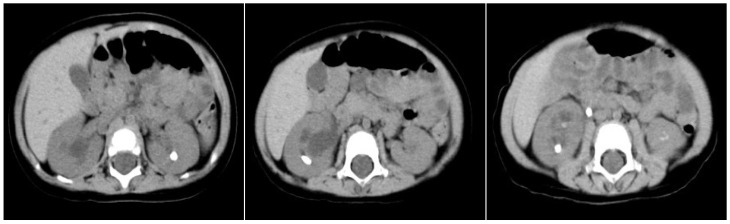
Computed tomography scan image of the abdomen.

An 8-month-old infant was admitted to the hospital due to fever. Oral antibiotics were prescribed; however, the fever persisted. The patient had no other health problems during the perinatal period. Blood tests showed an elevated inflammatory response: CRP 5.15 mg/dL (<0.5), WBC 26,200/µL (4500–9000), Ca 11.3 mg/dL (8.9–11.8), iP 5.7 mg/dL (3.1–7.9), and Intact PTH 8.3 pg/mL (15–50). Urine tests showed urine blood (+-), urine protein (+-), WBC 30–49 per high-power field (HPF), negative urine culture, urinary Ca 0.9 mg/dL (7–20), urinary Cr 10.5 mg/dL (67–100), and urinary Ca/Cr 0.086 (<0.81). The fever was resolved promptly after cefpirome administration. However, abdominal ultrasonography revealed multiple urinary tract stones in both kidneys, prompting a computed tomography (CT) scan. The CT scan confirmed multiple stones in both kidneys and ureters, along with right hydronephrosis (Figure 2). Subsequently, the patient underwent extracorporeal shock wave lithotripsy (ESWL). The urinary tract stones were found to be predominantly composed of calcium oxalate by infrared absorption spectrophotometry.

The patient continued to develop recurrent urinary tract stones and underwent ESWL at the ages of 9 months, 10 months, 1 year and 8 months, 4 years and 1 month, 4 years and 3 months, and 4 years and 5 months. He underwent transurethral lithotripsy at the age of 5 years and 4 months. Subsequently, he required only one additional transurethral lithotripsy procedure and has not experienced significant lithiasis since. At present, he is 18 years old. An analysis of the mutations could not be performed because parental consent was not obtained.

### 2.2. Sample Preparation and GC/MS Analysis

(4*R*)-4-Hydroxy-L-glutamate was obtained from Sigma Aldrich (St. Louis, MO, USA). Sample preparation and GC/MS-based urine metabolomics were performed as described previously [7]. The urine samples (100 μL) were pretreated with type C-3 urease at 37 °C for 10 min to remove urea interference and then spiked with internal standards, including 100 nmol of [^2^H_3_] creatinine and 25 nmol of 2,2-dimethylsuccinate. Protein, including the added urease, was precipitated with ethanol and removed. The deproteinized solution was evaporated to dryness, and compounds of interest in the dried residue were converted to trimethylsilyl (TMS) derivatives and analyzed using GC/MS. During this procedure, creatine is converted to creatinine. Thus, the creatinine concentration obtained by the current procedure represents “total creatinine” (i.e., creatinine plus creatine) and was quantified using D3-creatinine as the internal standard.

Aliquots (1 μL) of the derivatized extracts were injected into an Agilent gas chromatograph (7890B GC system; Santa Clara, CA, USA) mass spectrometer (5977B MSD) equipped with a fused-silica DB-5 capillary column (30 m × 0.25 mm id. with a 0.25-μm film thickness; J&W, Folsom, CA, USA) with a split ratio of 1:10 to 1:30. The oven temperature was programmed to increase from 60 °C to 320 °C at a rate of 17 °C/min. Electron impact mass spectra were obtained at a scan rate of 4.7 cycles/s from *m*/*z* 50 to 650.

## 3. Results

### Urinary Metabolomics

**Scheme 1:**
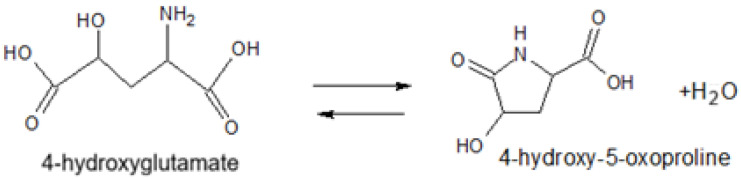
Chemical conversion of 4-hydroxyglutamate, 4HGlu, to 4-hydroxy-5-oxoproline, 4HGL, during the sample processing employing urease.

**Figure 3:**
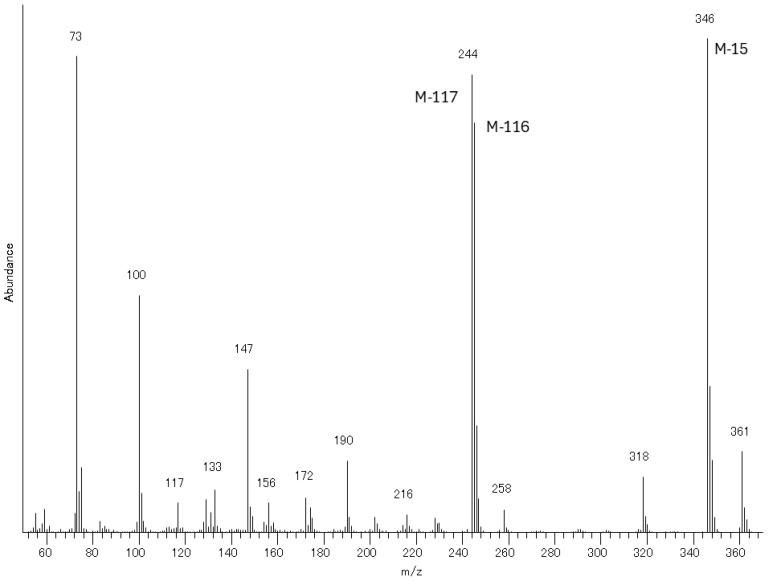
Mass spectrum of 4-hydroxy-5-oxoproline, 4HGL, formed by lactonization of 4-hydroxyglutamate.

**Figure 4:**
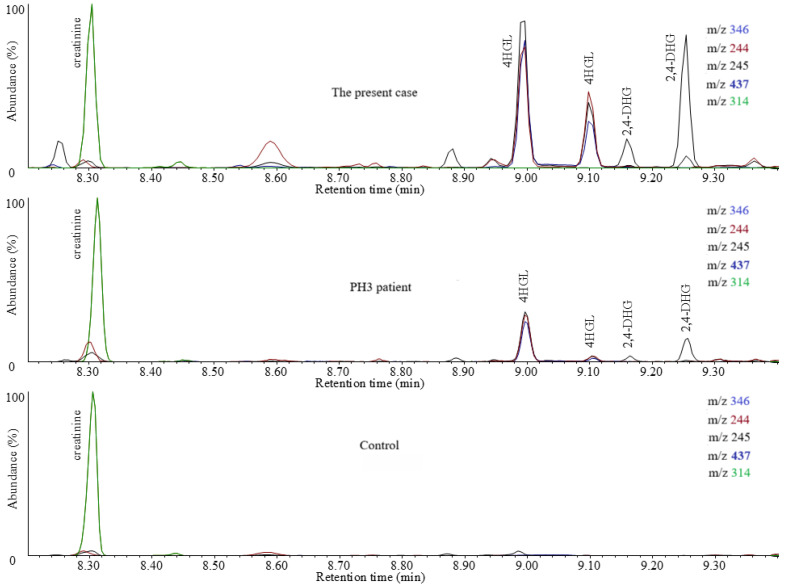
Extracted ion chromatograms of urine metabolome, normalized to creatinine. **Top**: eight-month-old Japanese patient in the present study; **middle**: six-year-old genetically confirmed case with PH3; and **bottom**: ten-month-old healthy Japanese control. 4HGL: 4-hydroxy-5-oxoproline, i.e., 4-hydroxyglutamate lactone, formed by lactonization of 4-hydroxyglutamate during sample preparation. 2,4-DHG: 2,4-dihydroxyglutarate.

We previously obtained the metabolome data of the spot urine at the age of 8 months and 26 days by using GC/MS, as described previously [9]. This step was performed nine years prior to the discovery of PH3 [1,2,3], and the cause of this child’s stone disease was unknown at the time. We retrospectively analyzed the previously acquired metabolome data and confirmed that the patient had PH3. The stable PH3 biomarker 4HGlu is converted to two isomers of the lactone, 4HGL, during sample preparation involving urease treatment, which was confirmed using (4*R*)-4-hydroxy-L-glutamate (Scheme 1); the mass spectrum of 4HGL is shown in Figure 3. These lactones were markedly increased in the patient’s urine (Figure 4, top) and were quantified as 99 μmol/mmol creatinine.

**Figure 5:**
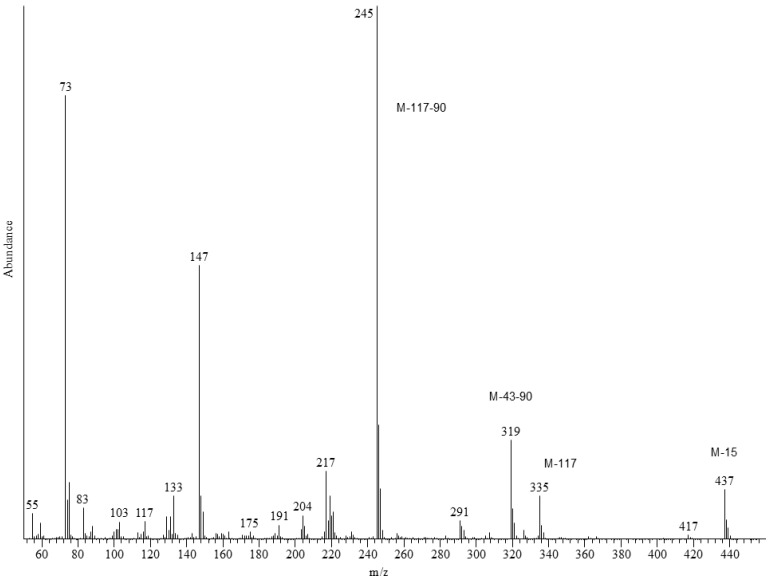
Mass spectrum of trimethylsilyl derivative of 2, 4-dihydroxyglutarate, 2,4-DHG.

Another PH3 biomarker, 2,4-DHG, exists as diastereomers and therefore forms two peaks. The mass spectrum is shown in Figure 5. A significantly increased level of 2,4-DHG was observed in the patient’s urine (Figure 4, top).

The simultaneous increase in 4HGL and 2,4-DHG was unique to this patient’s urine sample and has not been observed in over 5000 evaluated individuals, including extensive controls, and no alternative conditions producing this profile are known (Figure 4, bottom). Only one Australian case could be compared, in which the disease was diagnosed upon the detection of *HOGA1* mutations and conducting a metabolic study using electrospray ionization tandem mass spectrometry (Figure 4, middle). The PH3 biomarker 4-hydroxy-2-oxoglutarate was undetectable, possibly because of instability during sample preparation. The oximation of samples may improve the detection of this metabolite. Glycolate and glycerate, the critical biomarkers of PH1 and PH2, respectively, were not increased.

Although 2,4-DHG was not detected in controls, a moderate increase in 4-HGlu was observed in a few patients with primary hyperammonemia; in these cases, however, orotate, uracil, uridine, glutamine, and additional analytes served as biomarkers to distinguish primary hyperammonemia from PH3 [14].

## 4. Discussion

Conventional urine organic acid profiling using GC/MS has been previously employed to diagnose PH3. However, detection of 2,4-DHG is inefficient because of the compound’s high polarity and poor extraction by commonly used solvents, typically ethyl acetate, which is not appropriate for PH3 screening. In contrast, the urease method applied in the present report offers the advantage of detecting the biomarker 2,4-DHG with higher sensitivity. In our patient, 2,4-DHG was significantly elevated, whereas it was undetectable in other patients or controls. Notably, Woodward et al. screened for PH3 by targeting 2,4-DHG using liquid chromatography–tandem mass spectrometry and reported that all screening-positive cases were subsequently found to have pathogenic *HOGA1* mutations [7]. The levels of 4HGlu, the other critical biomarker, were reported to decline steadily with age; therefore, the use of age-matched controls is recommended [6]. In the same report, the 4HGlu level in patients with PH3 ranged from 6.5 to 98 μmol/mmol creatinine upon using flow injection tandem mass spectrometry. 4HGlu is almost completely converted into two isomers of the lactone 4HGL during sample preparation with urease treatment, allowing for the detection of 4HGlu as 4HGL with high sensitivity. In the present case, the 4HGlu in the urine was 99 μmol/mmol creatinine when the child was as young as 8 months and 26 days of age.

Regarding the increase in 4-HGlu in a few patients with primary hyperammonemias, one possible mechanism is that excess ammonia lowers the amount of mitochondrial α-ketoglutarate necessary for transamination of 4-HGlu to 4-hydroxy-2-oxoglutarate, thus favoring an increase in 4-HGlu. To our knowledge, no reports have suggested a change in 2,4-DHG levels in any other diseases [15]. Confirming the simultaneous increase in both biomarkers appears to be more accurate. It remains possible that under certain pathological conditions, one or both biomarkers may increase in the future. Nevertheless, GC/MS-based metabolomics provides broad chemical coverage. Unlike neonatal mass screening, high-risk screening is supported by detailed clinical information and laboratory findings. Comprehensive metabolomic profiling, encompassing hundreds of analytes, together with these clinical data, is expected to allow for reliable differentiation from PH3.

The clinical course of PH3 is considered less severe than that of other PH subtypes; however, the late diagnosis of a 78-year-old man who developed renal failure after a 30-year history of urolithiasis suggests that PH3 may be underdiagnosed in adults and elderly patients [16]. In another case, kidney failure developed at 33 years of age [17]. Warnings issued in 2015 noted that renal function could be impaired even in childhood [18]. Notably, PH3 patients exhibit a median age of first symptoms of 2.7 (0.9, 8.7) years, compared with 4.9 (1.7, 13.6) years for PH1 and 5.7 (1.4, 15.2) years for PH2 [19]. A recent OxalEurope cohort study demonstrated that PH3 is not a benign condition, but rather manifests as early-onset, recurrent stone disease and can impair kidney function [20]. A high prevalence of PH3 was also identified in a unique cohort with variable stone disease severity and degrees of chronic kidney disease [21]. Recent research conducted by two consortia demonstrated that a large proportion of individuals who are at risk for PH symptoms remain undiagnosed [5]. To address this issue, improved screening and diagnostic strategies for PH are considered essential; however, the diagnostic criteria for PH have not been verified [22]. Earlier diagnosis and intervention are desired to contribute to better outcomes for PH1 patients in Japan [23]. Although urinary biomarker analysis can provide a definitive diagnosis of PH1 within days, subsequent confirmation of genetic mutations often requires years [24]. In contrast, all PH2 cases we identified as PH2 through urinary metabolomic profiling were subsequently confirmed by mutation analysis relatively simply compared to PH1 cases [10].

Mutation analysis is widely and rapidly expanding [5,25,26] and may become readily available at a low cost in the near future. However, a subset of patients will inevitably decline genetic testing because of concerns regarding the disclosure of incidental or unwanted genetic information. The current standard diagnostic criteria rely on mutation analysis or enzymatic demonstration through biopsy, which is an invasive method and currently rarely performed. If mutation analysis cannot be conducted because of a lack of consent as in this case, the diagnosis remains uncertain because it does not align with the existing criteria. For hereditary urolithiasis, substantial biological knowledge has accumulated with globally validated reliability. For example, 2,8-dihydroxyadenine lithiasis has had a long history since its discovery in 1957 [27], and its metabolism, biomarkers, and APRT mutations have been extensively evaluated [28,29,30,31]. Although effective treatments are available, APRT may be diagnosed several years or even decades after symptom onset. Molecular analysis of metabolites using chromatography–mass spectrometry can detect quantitative and qualitative changes that reflect the presence of pathogenic mutations, regardless of whether such mutations are known or novel, common or rare, or located within exons or introns. Therefore, this technique may serve as a diagnostic criterion offering a non-invasive alternative to enzymatic testing, provided that each facility ensures complete documentation of both the analytical method and patient-specific confidence levels in its classifications. The adaptation of new diagnostic criteria holds immense value for undiagnosed patients.

The GC/MS metabolomics approach can detect PH1 [24] and PH2 [10], PH3, APRTD [11,12], and xanthinuria [13] in a single analysis through simultaneous evaluation of metabolites that are critical biomarkers for each IEM: glycolate for PH1; glycerate for PH2; 4HGL and 2,4-DHG for PH3; adenine, 8-hydroxyadenine and 2,8-dihydroxyadenine for APRTD; and hypoxanthine, xanthine, urate, and hydantoin-5-propionate for xanthinuria.

## 5. Conclusions

To the best of our knowledge, this is the first report of a Japanese patient with PH3. However, based on the current criteria, the diagnosis in this case remains uncertain. The simultaneous increase in the levels of two biomarkers, 2,4-DHG and 4-HGlu, together with the presence of early-onset recurrent urinary tract stones, predominantly comprising calcium oxalate deposits, strongly supports the diagnosis of PH3. A moderate increase in 4HGlu was observed in a few patients with primary hyperammonemia. However, GC/MS-based metabolomics enable us to distinguish PH3 from primary hyperammonemias. GC/MS-based urine metabolomics is non-invasive, rapid, and low-cost and provides molecular-level diagnostic information from spot urine. This technique can also be used to monitor disease progression, as patients with PH3 benefit from long-term follow-up, particularly during the transition from childhood to adulthood. The application of metabolite-level diagnosis by using these molecular analytical techniques as a diagnostic criterion would be an innovative step that would benefit many undiagnosed patients, their families, and society.

## Data Availability

Data are available from the corresponding author upon reasonable request.

## Abbreviations

The following abbreviations are used in this manuscript:

PH	primary hyperoxaluria
GC/MS	gas chromatography/mass spectrometry
IEM	inborn errors of metabolism
HOGA	4-hydroxy-2-oxoglutarate aldolase
2,4-DHG	2,4-dihydroxyglutarate
4HGlu	4-hydroxyglutamate
4HGL	4-hydroxy-5-oxoproline
ESWL	extracorporeal shock wave lithotripsy
TMS	trimethylsilyl
